# The adenoma-carcinoma sequence in the colorectum--early appearance of a hierarchy of small intestinal mucin antigen (SIMA) epitopes and correlation with malignant potential.

**DOI:** 10.1038/bjc.1992.351

**Published:** 1992-10

**Authors:** S. J. Pilbrow, P. J. Hertzog, A. W. Linnane

**Affiliations:** Biochemistry Department, Monash University, Clayton, Victoria, Australia.

## Abstract

**Images:**


*Br. J. Cancer* (1992), **66**, 748–757

© Macmillan Press Ltd., 1992

### The adenoma-carcinoma sequence in the colorectum – early appearance of a hierarchy of small intestinal mucin antigen (SIMA) epitopes and correlation with malignant potential

S.J. Pilbrow[1], P.J. Hertzog[2], & A.W. Linnane[1,2].

[1]*Biochemistry Department, Monash University, Wellington Road, Clayton, Victoria, 3168;* [2]*Center for Molecular Biology and Medicine, Monash University, Clayton, Victoria, 3168, Australia.*

**Summary** The colorectal adenoma-carcinoma sequence was examined in relation to the ectopic expression of the oncofoetal Small Intestinal Mucin Antigen (SIMA), to the development of morphologic changes in the adenoma and perineoplastic mucosa and to indices of malignant potential. Four anti-SIMA MAbs, which define a novel hierarchy of SIMA epitopes in the normal small intestine and adjacent to colorectal cancers, were used in a retrospective immunohistochemical study of Familial Adenomatous Polyposis (FAP, $n = 183$) and non-familial ($n = 44$) adenomas. Inappropriate expression of SIMA epitopes was first detected in mucosa adjacent to minute microadenomas larger than three glands, and with increase in size, in increasing amounts within adenomas themselves, but not with microadenomas smaller than three glands or regions of flat mucosa free of adenomas. SIMA epitope expressed in mucosa adjacent to adenomas preceded changes in perineoplastic morphology, which progressed with adenoma growth to resemble transitional mucosa (TM) adjacent to cancers. Thus, the onset of both SIMA expression and morphological changes in TM were consistent with reactive rather than pre-existing field change phenomena. The previously reported hierarchy of four SIMA epitopes (5C5, 3D4, 4D3, 6C5) was also consistently observed in the adenoma-carcinoma sequence, and applied to (i) the order of epitope detection, (ii) the number of positive adenomas and (iii) extent of staining; (iv) the height in the crypt and (v) distance from the adenoma to which epitopes were expressed in perineoplastic mucosa. These observations are consistent with a progression of changes in mucin composition with adenoma development. The percentage of positive adenomas and reactivity scores for each anti-SIMA MAb correlated with increasing adenoma size, degree of dysplasia and growth pattern. SIMA expression appears to predate the earliest reported oncogene and tumour suppressor gene changes, was persistent and increased throughout adenoma development. SIMA epitopes are thus markers of very early neoplastic change, whose expression correlates with malignant potential and may contribute to the accumulation of changes necessary for tumourigenesis.

The adenoma-carcinoma sequence in the colorectum is strongly supported by epidemiological data (Enterline *et al.*, 1976; Hill *et al.*, 1978), genetic studies (Cannon-Albright *et al.*, 1988), pathological data (Muto *et al.*, 1975) and the natural history of untreated familial and non-familial adenomas (Jackman & Mayo, 1951; Stryker *et al.*, 1987). The progression from adenoma to carcinoma has been proposed to be a multi-step process (Hill *et al.*, 1978) and activation of particular proto-oncogenes and inactivation of tumour suppressor genes have recently been identified at particular stages in the evolving adenoma (Fearon & Vogelstein, 1990).

Other important molecular changes associated with colorectal neoplasia include alterations in mucin composition, not only in cancers themselves (Feizi *et al.*, 1984; Bara *et al.*, 1980; Hertzog *et al.*, 1991*a*,*b*), but also in the adjacent, non-neoplastic 'transitional mucosa' (TM) (Filipe, 1979; Bara *et al.*, 1984; Pilbrow *et al.*, 1992*a*). It has been proposed that TM represents a pre-existing field change (Filipe, 1979) although other studies suggest a reactive change (Isaacson & Attwood, 1979; Listinsky & Riddell, 1981). The former contention has gained support from descriptions of similar morphologic, histochemical and antigenic changes in premalignant epithelium, namely ulcerative colitis (Podolsky *et al.*, 1983; Filipe *et al.*, 1988), adenomatous polyps (Filipe *et al.*, 1980; Bara *et al.*, 1983; Itzkowitz *et al.*, 1986) and animal models of colon carcinogenesis (Filipe, 1975; Decaens *et al.*, 1983). However, whether mucin and TM changes in the human adenoma-carcinoma sequence represent a field change or a reactive change remains controversial.

In previous studies from our laboratory, we have produced new MAbs to gastrointestinal mucins, SIMA (Small Intestinal Mucin Antigen), and LIMA (Large Intestinal Mucin Antigen). SIMA, an oncofoetal antigen of the colon, distinct from the normal colonic antigen LIMA, is a high molecular weight mucin glycoprotein with repeating epitopes, expressed inappropriately in 94% colorectal cancers (Hertzog *et al.*, 1991*a*,*b*). Using a panel of four anti-SIMA MAbs (5C5, 3D4, 4D3 and 6C5), we recently demonstrated a novel hierarchy of epitope expression in the normal small intestine villus, which was reexpressed in TM crypts adjacent to colorectal cancers (Pilbrow *et al.*, 1992*a*). A comparable hierarchy of three LIMA epitopes in the normal colon crypt has also been reported (Pilbrow *et al.*, 1992*b*). In a study which mapped the extent of changes in Swiss Roll preparations of perineoplastic mucosa around colorectal cancers, SIMA was detected in adjacent TM in 15/15 cases, and in distant morphologically normal mucosa at the resection margins in 11/15 cases (Pilbrow *et al.*, 1992*a*). Whether such epithelial changes were pre-existing or reactive to the cancer was unknown. In order to address this question, we have used this same panel of four anti-SIMA MAb to examine changes in mucin composition and perineoplastic morphology in the human adenoma-carcinoma sequence using adenomas of a wide range of sizes. degree of dysplasia and growth pattern, three well-established indices of malignant potential (Muto *et al.*, 1975).

### Materials and methods

### *Source of tissue specimens and clinical information*

Retrospective paraffin blocks and/or newly resected adenoma specimens were obtained, including where possible, several cm of adjacent mucosa. Small and large intestinal specimens from patients free of GI tract disease were used as positive/negative tissue controls. Pathologist's reports detailing size,

Correspondence: S.J. Pilbrow.
Received 10 December 1991; and in revised form 21 May 1992.

degree of dysplasia and growth pattern accompanied each specimen:

*(i) FAP (nine patients, 208 adenomas, Table I)* Two were male, seven female, mean age 23.9 (range 15–40). Adenomas were from sites throughout each colectomy specimen, in most cases unspecified. Two patients had a synchronous cancer.

*(ii) Non-familial adenomas (32 patients, 44 adenomas, Table I)* Ten patients were male (mean age 61.2, range 43–73), ten female (mean age 66.8, range 59–77), and 12 of unspecified age and sex. Anatomical sites were caecum (2), asc. colon (9), hep. flexure (2), trans. colon (2), desc. colon (2), sigmoid (5), rectum (7), unspecified (15). Six cases had synchronous cancer; one had ulcerative colitis.

*(iii) Metaplastic polyps (ten patients, 27 polyps)* These included five male, five female (mean age 66.3, range 57–87). Three patients had synchronous colorectal cancers, one of whom had 13 metaplastic polyps. (Additional polyps within the TM zones of the cancers were excluded).

### *Histopathology*

Specimens were fixed in phosphate buffered formalin, paraffin-embedded, and cut into serial 5 μm sections. One section per block was routinely stained using the Alcian Blue (pH2.5)/periodic acid-Schiff/Mayer's Haematoxylin stain, projected at a 10 × magnification and tissues outlined. Adenomas were identified, and dimensions measured. From these, and in larger cases, from dimensions in the pathologists' reports, approximate cross-sectional area (length × height) (Goh and Jass, 1987) was calculated for each adenoma, and thereby grouped into semi-log sizes ($<0.1$ $mm^2$, 0.1–0.3 $mm^2$, 0.3–1.0 $mm^2$, . . . 3,000–10,000 $mm^2$, Table I). In the three smallest size groupings, adenoma dimensions were measured using a stage micrometer, and the cross-sectional number of glands in each adenoma was counted for comparison with surface area [$<0.1$ $mm^2$: 1–4 glands, median three glands ($n$ = ten adenomas); 0.1–0.3 $mm^2$: 3-18 glands, median seven glands ($n = 21$); 0.3–1.0 $mm^2$: 9–42 glands, median 20 ($n = 37$)]. Assessments were made of degree of dysplasia (mild, moderate, severe) and growth pattern (tubular, tubulovillous, villous) by author SJP according to current WHO criteria (Jass & Sobin, 1989).

Adenomas of both groups were of similar morphology, although those from the younger FAP group were predominantly smaller, less dysplastic and more tubular than the non-familial adenomas (generally larger, mor dysplastic, greater villous component). In both groups the degree of dysplasia and growth pattern were partly related to size (Table I). Finally, zones of altered morphology in adjacent mucosa were characterised. 25/208 FAP microadenomas identified within the 'shadow' of abnormal mucosa adjacent to larger adenomas were excluded from the study, leaving 183 'independent' adenomas.

### *Monoclonal antibodies*

The production, immunochemical and immunohistochemical characterisation of the MAbs used in this study have been described previously. Anti-SIMA MAb 4D3 and anti-LIMA MAb 2C3 were prepared by immunisation of mice with a mucin extract from a colorectal cancer (Hertzog *et al.*, 1991*a* and *b*), and anti-SIMA MAb 3D4, 5C5 and 6C5 by immunisation with a mucin extract of post-mortem tissue from normal adult small intestine (Pilbrow *et al.*, 1992*a*). Hybridoma supernatants were stored frozen at −20°C, and thawed at room temperature immediately prior to use. All four anti-SIMA MAbs were shown previously to be neuraminidase- and periodate-sensitive, and to recognise different epitopes in ELISA and immunohistochemical studies, distinct from Sialosyl-Tn (MAb TKH2). MAb isotypes were $IgG_3/\kappa$ [4D3]; $IgG_1/\kappa$ [6C5,2C3]; $IgM/\kappa$ [3D4,5C5] (Hertzog *et al.*, 1991*a*; Pilbrow *et al.*, 1992*a*).

### *Immunohistochemistry*

Consecutive 5 μm sections of each block were dewaxed, reacted with each of the five MAbs, stained by an indirect immunoperoxidase technique (Hertzog *et al.*, 1991*a*), and counterstained with Haematoxylin. Working dilutions for each of the primary and secondary antibodies were established by checkerboard titrations using normal colon and small intestinal tissue controls. Horseradish peroxidase (HRP)-conjugated rabbit anti-mouse immunoglobulin (Ig) was obtained from Dakopatts (Denmark).

For each MAb, a qualitative assessment of the predominant cellular staining patterns was made. For each adenoma, reactivity patterns of the five MAb were recorded

**Table I** Classification of adenomas to size, degree of dysplasia and growth pattern by number of adenomas

| | *Tubular* | | | *Tubulovillous* | | | *Villous* | | | |
|---|---|---|---|---|---|---|---|---|---|---|
| | *Mild* | *Mod* | *Sev* | *Mild* | *Mod* | *Sev* | *Mild* | *Mod* | *Sev* | *Total* |
| Area ($mm_2$) | | | | | | | | | | |
| <0.3 | **32** | | | | | | | | | **32** |
| | 1 | | | | | | | | | 1 |
| 0.3–1.0 | **36** | **1** | | | | | | | | **37** |
| | | | | | | | | | | 0 |
| 1.0–3.0 | **39** | **2** | | | | | | | | **41** |
| | 0 | | | | | | | | | 3 |
| 3.0–10 | **26** : | **8** | | | | | | | | **34** |
| | 3 | 1 | | 1 | | | | | | 5 |
| 10–30 | **7** | **7** | | **3** | **1** | | | | | **18** |
| | 1 | 1 | | 1 | 1 | | | | | 4 |
| 30–100 | **1** | **8** | | **1** | **3** | **2** | | **1** | | **16** |
| | | 1 | | 1 | 2 | | | 1 | 1 | 6 |
| 100–300 | | **1** | | **2** | **1** | | | | | **4** |
| | | 1 | 1 | 4 | 1 | 1 | | 4 | 2 | 14 |
| 300–1000 | | | | | | | | | | **1** |
| | | 1 | 1 | | 1 | 3 | | 1 | 1 | 7 |
| >1000 | | | | | | | | | | **0** |
| | | | | | 1 | | 1 | 2 | | 4 |
| Total | **141** | **28** | | **6** | **5** | **2** | | **1** | | **183** |
| | 8 | 4 | 2 | 7 | 6 | 4 | 1 | 8 | 4 | 44 |

Key: **FAP, bold type, upper line**; *Non-familial adenomas*, normal type, lower line, *Degree of dysplasia* [mild, mod (moderate), sev (severe)]; *growth pattern* [tubular, tubulovillous, villous]; *size* (surface area $mm_2$).

in three areas where present: neoplastic adenoma tissue, base of adenoma and immediately adjacent perineoplastic mucosa. A previously described scoring method was used: a score of 0–3 was given for staining intensity and similarly, for distribution [0 (negative, 1 (<25% of crypt positive), 2 (25–75%) or 3 (75–100%)]. Aggregate scores (0–6) were obtained by adding the two scores (Hertzog *et al.*, 1991*b*).

*Data analysis*

Univariate correlations were made between each individual MAb reactivity (whether positive or negative; and reactivity scores) in the adenoma, base and adjacent mucosa and the three indices of malignant potential: size (surface area), degree of dysplasia and growth pattern, for both FAP and non-familial groups. Scores were expressed as mean ± standard error of mean (SEM) as a measure of variance of these scores in our patient population. Perineoplastic morphological zones were also correlated with adenoma size.

### Results

A. *Qualitative immunohistochemical reactivity patterns*
*(i) SIMA expression in the developing adenoma*

In the smallest, single gland microadenomas, there were generally no SIMA epitopes expressed at all (Figure 1a). In microadenomas greater than approximately three glands in size, traces of SIMA were identified adjacent to but not in the actual neoplastic adenoma glands. With increasing size, SIMA expression was more prominent at the base of the adenoma and in the immediately adjacent perineoplastic mucosa, and detectable in neoplastic adenoma glands (Figure 1b and 1c). With larger adenomas, SIMA was expressed to an increasingly greater extent in the neoplastic adenoma glands, beginning in the inner, least dysplastic areas (Figure 1d), and eventually involving the entire adenoma, including distal villous areas (Figure 1e) and the most severely dysplastic areas (Figure 1f). Specific patterns of individual epitopes are discussed in detail below (section A(iv)). Similar to

**Figure 1** Immunoperoxidase staining of sections of adenomas at various stages in development with anti-SIMA MAb 4D3: **a**, a single gland microadenoma (arrow) showing no reactivity (original magn. × 25); **b**, a larger microadenoma showing patchy goblet cell and secreted mucin reactivity in non-neoplastic glands within the substance of the adenoma (arrow) and at the base and in adjacent mucosa (arrowheads) (original magn. × 10). In larger adenomas, strong staining is shown both **c**, at the base of the adenoma (goblet cells, arrow) (original magn. × 25) and **d**, within neoplastic glands of adenoma (goblet cells, arrow) (original magn. × 25) **e**, the outer tip of a villous adenoma (goblet cells, arrow) (original magn. × 25), and **f**, a severely dysplastic area of a very large adenoma (secreted mucin, arrow) (original magn. × 25).

previous studies, the normal colonic anti-LIMA MAb 2C3 reacted with adenomas from the smallest (SIMA-negative) microadenomas to quite large adenomas, including areas which were expressing SIMA epitopes (Pilbrow *et al.*, 1992*b*).

*(ii) Localisation at the cellular level* In and adjacent to adenomas, each anti-SIMA MAb reacted with goblet cell (GC) and extracellular secreted mucin (Figure 3a); these are the same cellular compartments as previously reported for normal small intestine, colorectal cancer and adjacent TM (Hertzog *et al.*, 1991*a*,*b*; Pilbrow *et al.*, 1992*a*). Each anti-SIMA MAb reacted also with mucinous lakes in large adenomas, and in some cases, with serum in submucosal blood vessels. Anti-LIMA MAb 2C3 reacted with GCs and extracellular mucin in small to moderately-sized adenomas, similar to normal colon; with increasing adenoma size, extracellular mucin staining persisted, apical staining became more prominent, and GC reactivity diminished, resembling its pattern in cancers (Pilbrow *et al.*, 1992*b*).

*(iii) Crypt localisation* In smaller adenomas, anti-SIMA MAb reactivity was strongest at the base of the dysplastic 'crypts' (the innermost part) of adenomas (Figure 1b), and in the lower crypt of the perineoplastic mucosa (Figure 2a). In larger adenomas, anti-SIMA reactivity was increasingly widespread throughout the adenomas (Figure 1d) and to a greater height in the crypt of the immediately adjacent mucosa (Figure 2b), but reducing to a lower portion of the crypt with increasing distance; this was similar to the pattern adjacent to cancers (Pilbrow *et al.*, 1992*a*). Anti-LIMA MAb 2C3 was generally reactive throughout smaller adenomas and their perineoplastic mucosa; within larger adenomas, it reacted more patchily throughout but remained strongly reactive in perineoplastic crypts.

*(iv) Comparison between individual anti-SIMA MAbs (Figure 3)* Most of the smallest microadenomas expressed no SIMA epitopes, although some expressed the 5C5 epitope. With increase in adenoma size, additional epitopes were detected in a consistent sequence: the pattern of 'recruitment' of epitopes detected appeared to be 5C5, 5C5 + 3D4, 5C5 + 3D4 + 4D3, and eventually 5C5 + 3D4 + 4D3 + 6C5 in large adenomas. Alternative combinations were rare, and 6C5 was essentially only expressed in adenomas which also expressed the other three SIMA epitopes. Each MAb followed the same apparent order of tissue localisation with increase in adenoma size: base, adjacent, inner core of adenoma, throughout adenoma, although MAb 5C5 showed more prominent reactivity with actual adenoma glands in microadenomas.

B. *Semi-quantitative and clinicopathological correlations*

*(i) Percentage of positive adenomas* The percentage of adenomas reactive with each anti-SIMA MAb correlated with each of the three indices of malignant potential: increasing size (Figure 4), degree of dysplasia (Figure 5) and progression from tubular to villous growth pattern (Figure 6). Throughout most size ranges, three of the anti-SIMA MAbs (4D3, 3D4 and 6C5) were more frequently reactive in the base of the adenoma than in the adjacent tissue and least in the actual adenoma. This was most apparent in the size ranges from the 0.3–1.0 $mm^2$ range (9–42 glands, median 20 glands, $n = 37$) to the 10–30 $mm^2$ range (Figure 4). In contrast, MAb 5C5 generally showed prominent reactivity at all sizes in both the adenoma, base and adjacent mucosa, even the smallest ones. In adenomas with a cross-sectional area of greater than 100 $mm^2$ (i.e. diameter $>$ 1 cm), 100% of FAP adenomas reacted with all anti-SIMA MAbs (Figure 4). For each index, 5C5 was the most frequently detected in lower grades of malignant potential (small, mildly dysplastic, tubular), followed by 3D4, 4D3 and 6C5; in the highest grades (large, severely dysplastic, villous) all 4 epitopes were detected. Nonfamilial adenomas showed similar correlations between SIMA epitopes and indices of malignant potential (Figure 5). SIMA showed no association with local

**Figure 2** Immunoperoxidase staining of MAb 4D3 in perineoplastic mucosa: **a**, adjacent to small adenomas, in lower 1/3 of crypt and **b**, adjacent to large adenomas throughout crypt (goblet cells, arrows) (original magn. ×25).

**Figure 3** Comparison between immunohistochemical reactivities of four anti-SIMA MAbs in a small pedunculated adenoma (**a-d**) and its perineoplastic mucosa (**e-h**): 5C5 (**a** and **e**), expressed to the greatest extent in the adenoma and to the greatest distance from the adenoma; 3D4 (**b** and **f**) and 4D3 (**c** and **g**) expressed strongly at the base of the adenoma, and a short distance into the perineoplastic mucosa, 6C5 (**d** and **h**) expressed to a limited extent at the base only (original magnifications × 10).

inflammatory changes, which were observed only in relation to larger adenomas.

*(ii) Staining intensity and distribution* As a semi-quantitative measure of SIMA expression per unit area of tissue, mean reactivity scores (0–6) for each MAb were shown to correlate with size, dysplasia and growth pattern. Mean scores (± SEM) for MAbs 5C5 and 6C5 *vs* size are shown (Figure 7). Notably, MAb 6C5 showed only trace amounts until the 10–30 $mm^2$ size range, while MAb 5C5 reacted at much smaller sizes (<0.1 $mm^2$, approx. 3–4 glands).

C. *Perineoplastic mucosa*

*(i) Description of morphological zones* Five zones of perineoplastic mucosa were identified from marked atypia to morphologically normal mucosa (MNM), with features comparable to TM zones around colorectal cancers (Pilbrow *et al.*, 1992*a*): TM-I, a very small zone of few small GCs and many columnar cells at the adenoma base (Figure 8a); TM-II, elongated, dilated and branched crypts, croweded with large goblet cells (GC) (Figure 8b); Zone III, crypts of normal height but dilated and with enlarged GCs (Figure 8c); Zone IV, patchy crypt dilatation and scattered large GCs interspersed with normal colorectal morphology; and MNM (Figure 8d).

*(ii) Relationship between morphological zones and adenoma size* Zones of increasing atypia, as defined above, appeared to be sequentially acquired with increasing FAP adenoma size (Figure 9). The smallest microadenomas (<0.1 $mm^2$, 1–4 glands; median 3)] were all surrounded by MNM only. However, in the 0.1–0.3 $mm^2$ range (3–18 glands; median 7), 53% showed minor TM changes (zone IV, 48%; zone III, 5%), and in the 0.3–1.0 $mm^2$ range (9–42 glands; median 20), 81% (zone IV, 32%; zone III, 49%). At each size, the most atypical zone was innermost, frequently interdigitating

**Figure 4** Percentage of FAP adenomas reacting with anti-SIMA MAbs as a function of size (surface area) in adenoma, base and adjacent mucosa: **a**, 5C5 **b**, 3D4 **c**, 4D3 and **d**, 6C5.

**Figure 6** Percentage of FAP adenomas reacting with all four anti-SIMA MAbs as a function of pattern of growth.

**Figure 7** Semiquantitative correlation of anti-SIMA MAb 5C5 and 6C5 reactivity scores (0–6) with adenoma size. Data are presented as mean ± S.E.M.

**Figure 5** Percentage of adenomas reacting with anti-SIMA MAbs 5C5 and 6C5 as a function of degree of dysplasia: comparison of FAP and non-familial adenomas.

with the adenoma, followed by concentric zones of diminishing atypia (e.g. adenoma, III, IV, MNM) similar to TM of colorectal cancers (Pilbrow *et al.*, 1992*a*). Zone I, only several crypts wide around cancers, was found only in isolated patches at the adenoma-perineoplastic interface of large adenomas. The length of each zone and the total length of TM increased with adenoma size, and appeared symmetrical proximal and distal to each adenoma.

Most non-familial adenomas showed TM changes similar to the FAP group, although, due to the more limited size range, the changes with increase in size were less clearly demonstrable. In addition, 5/17 of the adenomas $>100$ mm$^2$ showed submaximal TM changes .

*(iii) Expression of SIMA in perineoplastic zones* (Figure 8). With increase in adenoma size, SIMA expression in perineoplastic mucosa increased in intensity and was observed to a greater distance from the adenoma, and appeared symmetrical proximal and distal to each adenoma; in relation to the largest adenomas this resembled SIMA expression in TM adjacent to colorectal cancers. The maximum intensity for each adenoma was in the most atypical TM closest to the adenoma, but the intensity and extent of SIMA expression for a given zone and size of adenoma varied from one patient to another. When two adenomas were located close together, their SIMA-reactive zones sometimes overlapped (particularly with MAb 5C5, rarely with 6C5); when spaced widely apart, there were lengths of mucosa in between which did not express SIMA.

**Figure 8** Immunoperoxidase staining of perineoplastic mucosa zones adjacent to adenomas, using anti-SIMA MAb 5C5 **a**, TM-I with scanty small, unreactive GCs and mostly columnar cells (arrows), adjacent adenoma tissue (arrowhead); **b**, TM-II (tall, reactive goblet cells, arrow; dilated, mucin-filled crypt lumina, arrowhead); **c**, zone III with large, reactive goblet cells (arrow), less dilated crypts, and **d**, morphologically normal mucosa (MNM) with reactive GCs (arrow). (Original magnifications × 25).

There was frequently overlap of reactivity in GCs of the lower and middle crypt between the normal anti-LIMA MAb 2C3 and anti-SIMA MAbs.

In the cases where sufficient perineoplastic mucosa were available, the maximum distances to which each epitope was detected were measured. The most frequent pattern, seen in 17/24 FAP and 18/24 non-familial adenomas (see example in Figure 3), was that MAb 5C5 reacted to the longest distance (17 cm in one case), followed by 3D4 and 4D3, and least, 6C5, as observed with distance from cancers (Pilbrow *et al.*, 1992*a*). The mean ratios of maximum distance for each MAb to that of 5C5 in the 17 FAP adenomas showing this order were: 3D4/5C5, 0.64; 4D3/5C5, 0.64; 6C5/5C5, 0.23.

*Metaplastic polyps*

Of 27 metaplastic polyps (ranging from 0.25 to 100 $mm^2$ in area, mostly $<10\ mm^2$), trace amounts or more of SIMA epitopes were detected in GC/secreted mucin by MAb 5C5 (30% polyps: 0/13 from one patient, 8/14 remaining polyps), 3D4 (67%), 4D3 (52%), 6C5 (44%); 100% were LIMA (2C3) positive. In this small study, no trends were observed with respect to polyp size, nor differences between cancer- and non-cancer-bearing cases.

### Discussion

SIMA, an oncofoetal antigen for the colon, is established here as a marker of very early neoplastic change in the colorectum, common to both FAP and non-familial adenomas. By examining a more complete spectrum of adenoma size than in other studies to date, we were able to identify 4 SIMA epitopes in adenomas at earlier stages than studies of genetic changes (Fearon & Vogelstein, 1990) and altered expression of other mucin antigens (Zotter *et al.*, 1987; Wolf *et al.*, 1989). Altered SIMA expression commences before changes in perineoplastic morphology [transitional mucosa (TM)]; both changes follow microadenoma formation, evolve with adenoma development, and eventually

**Figure 9** The acquisition of morphological changes in perineoplastic mucosa with increase in size of FAP adenomas. Stacked bar graph shows maximum degree of morphological atypia observed in the immediate perineoplastic mucosa, as a relative percentage of adenomas, for each size range.

resemble those associated with colorectal cancer. We have further identified a differentiation-associated hierarchy of SIMA epitopes (Pilbrow *et al.*, 1992*a*) during the adenoma-carcinoma sequence, whose expression correlates with indices of malignant potential.

The adenoma-carcinoma sequence has recently been shown to be associated with the accumulation of specific genetic alterations: *ras* oncogene mutations, and allelic deletions of the 5q, 18q and 17p chromosomal regions proposed to contain tumour suppressor genes, such as the p53 gene (17p) (Fearon & Vogelstein, 1990) and the APC gene (5q21, Hoshino *et al.*, 1991). FAP patients have inherited mutations in the APC gene (5q21) (Groden *et al.*, 1991; Nishisho *et al.*, 1991); these can be detected in their polyps, whereas allelic losses of 5q have been described only in non-familial adenomas (only 3/8 <1 cm length adenomas, smallest 5 mm; Vogelstein *et al.*, 1988). The other genetic alterations however, are associated with more advanced adenomas: *ras* mutations with increasing size, villous pattern and dysplasia, and losses of 18q and 17p with advanced adenomas and malignant change respectively (Vogelstein *et al.*, 1988; Purdie *et al.*, 1991). However, our study of SIMA expression indicates that, over four orders of magnitude of surface area from 0.01 $mm^2$ (1-gland) microadenomas to 100 $mm^2$ (approx. 1 cm length) adenomas, additional events may be occurring. SIMA epitopes begin to be expressed in association with minute microadenomas (approximately 3-gland size), with the number of adenomas and quantitative SIMA reactivity increasing steadily with adenoma growth, their ordered appearance indicating a series of changes (see below). Moreover, at the 100 $mm^2$ size, when *ras* mutations and 5q deletions are still uncommon (Vogelstein *et al.*, 1988), all four SIMA epitopes are almost unversally expressed in adenomas of both groups. Furthermore, they continue to be highly expressed in cancers (Pilbrow *et al.*, 1992*a*). SIMA expression thus appears to be a very early and persistent change in the adenoma-carcinoma sequence, reflecting changes in differentiation, and which may itself contribute to the changes necessary for malignant change. By contrast, we have shown that the expression of normal colonic mucin (LIMA) epitopes, while prominent in GCs of small adenomas, decreases with increasing adenoma size (Pilbrow *et al.*, 1992*b*).

There was no field change predating the development of a tumour according to SIMA detection or morphological criteria. Trace amounts of SIMA were first noted at the base of the crypt of non-neoplastic MNM adjacent to very small microadenomas, later in the actual adenoma. This suggests that SIMA expression is a reactive change, possibly due to a factor secreted by the adenoma, exerting a paracrine effect initially on adjacent non-neoplastic cells but not on the tumour itself. Alternatively, a factor may be produced in a host response to the tumour. The detection of SIMA in increasingly peripheral regions of the adenoma with increase in size was similar to its detection in increasingly higher levels of the crypt in adjacent mucosa. This may reflect the upward expansion of the basal proliferative zone (Deschner, 1990), and the persistence of immature cells higher in the crypt and eventual loss of crypt polarity with adenoma development (Filipe, 1979).

The observation of the same order of SIMA epitopes (5C5, 3D4, 4D3, 6C5), as previously observed in normal small intestine and adjacent to cancers (Pilbrow *et al.*, 1992*a*), during adenoma progression, suggested a consistent order of changes in mucin composition. This applied to (i) the order in which SIMA epitopes first appeared adjacent to microadenomas; (ii) the number of positive adenomas <100 $mm^2$ size; (iii) the order in which epitopes were initially detected in microadenomas and later, the expansion of their expression throughout the adenoma, and (iv) the height in the colonic crypt and (v) maximum distance of expression in the perineoplastic mucosa, similar to the pattern observed around cancers (Pilbrow *et al.*, 1992*a*).

These results suggest that the small intestinal mucin phenotype we have previously observed in colorectal cancers is acquired gradually in the developing adenoma, in a similarly coordinated and sequential manner that reflects normal small intestinal enterocyte differentiation and crypt-villus relationships; this may represent a form of metaplasia (Agawa & Jass, 1990). In terms of epitope structure, previous studies have shown that the four anti-SIMA MAbs recognise distinct neuraminidase-sensitive carbohydrate epitopes, two which are distinct from sialosyl-Tn and 2 which partially cross-react (Hertzog *et al.*, 1991*a*; Pilbrow *et al.*, 1992*a*). Additive ELISAs have suggested that MAb 4D3 recognises a core carbohydrate structure, and the other three MAbs, more peripheral epitopes (Pilbrow *et al.*, 1992*a*). Precise characterisation of the epitopes is currently in progress.

While prospective studies show that only a minority of adenomas will ultimately undergo malignant change (Stryker *et al.*, 1987), three predictive indices of malignant potential are now well-established (Muto *et al.*, 1975). In the present study, SIMA expression, both in terms of numbers of positive adenomas and amount of epitope expressed, correlated positively with each of the three indices. Correlations have previously been reported between indices of malignant potential and altered expression of various blood group-related carbohydrate epitopes. Studies have reported normal proximal colon antigens reemerging in distal adenomas (Itzkowitz *et al.*, 1986), normal colonic antigens in inappropriate crypt and cell compartments (Ruggerio *et al.*, 1988), precursor accumulations (Cooper & Reuter, 1983) and neosynthesis of antigens (Kim *et al.*, 1986). The majority of these show altered or neo-expression late in adenoma development, in contrast to SIMA epitopes, however none has been examined systematically in minute adenomas to more precisely identify the onset of altered expression. Of neoexpressed mucin antigens, M1 was associated with villous (87%) more than tubular (55%) adenomas (Bara *et al.*, 1983), and MAM-6 (Zotter *et al.*, 1987) and TAG-72 (Wolf *et al.*, 1989) were only associated with severe dysplasia, thus also reflecting late changes.

The SIMA reactivity noted with serum in submucosal blood vessels of several larger adenomas suggests that at a particular stage of development, SIMA is released into the circulation. As several SIMA epitopes can be detected in sera of colorectal cancer patients (unpublished data), future retrospective and prospective studies will investigate at what stage SIMA is detectable in sera of adenoma patients and hence its potential clinical value.

Of interest was the fact that SIMA was also detected in metaplastic polyps, although no trends with respect to polyp size or synchronous colorectal cancer were observed. Notably, other oncofoetal antigens of the colon including T-antigen (Boland, 1987) have also been found in metaplastic polyps. While metaplastic polyps were long regarded as unrelated to neoplasia, recent evidence suggests clinically important associations with colorectal cancer (Jass, 1983; Teoh *et al.*, 1989; Foutch *et al.*, 1991), which may be reflected in common mucin biosynthetic/degradative pathways.

In addition to changes in mucin expression, significant observations were also made concerning the evolution of perineoplastic morphology during the adenoma development. The mucosa adjacent to adenomas has previously been noted to be similar histologically and histochemically to TM adjacent to cancers (Listinsky & Riddell, 1981; Filipe *et al.*, 1980), but whether it represents a pre-existing field change or a reactive change has remained controversial. Many studies of TM have been limited by technical problems and non-specificity of histochemical techniques, and varying definitions of TM morphology (Mughal & Filipe, 1978; Greaves *et al.*, 1984; Sugihara & Jass, 1987). Studies have also been limited by narrow ranges of adenoma size: predominantly large (Greaves *et al.*, 1984) or small (approx 20-gland; Schmidbauer & Heilmann, 1980), or unspecified size (Ruggerio *et al.*, 1988). The present study describes the sequential development of TM adjacent to adenomas over a wide range of sizes. Notably, TM changes were shown not to be a pre-existing field change, but to commence after microadenoma formation, at approximately a 7-gland size, and to evolve as concentric zones of increasing atypia.

The intensity of SIMA expression was greatest in the most atypical perineoplastic zones nearest the adenoma, lessening with increasing distance and decreasing morphological atypia, and absent in the morphologically normal flat mucosa between widely-spaced small adenomas. This failure to find SIMA expression in flat mucosa is in agreement with studies which found normal glycosyltransferase levels in flat mucosa of FAP patients (Slomski *et al.*, 1986) and some histochemical studies (Sugihara & Jass, 1987) but not others (Filipe *et al.*, 1980). This appears to be further evidence that mucin and perineoplastic morphological changes are only observed in relation to pre-existing adenomas. The present study would thus appear to have settled the longstanding debate: 'transitional mucosa: premalignant or reactive?' by identifying that altered mucin expression and TM morphological changes followed microadenoma formation, and are thus consistent with reactive change.

The authors wish to thank the following pathologists, Dr J. Pedersen (Alfred Hospital), Dr H. Preston (Caulfield General Hospital), Dr J. Dowling (Prince Henry's Hospital) and Dr D. Gee and Mr. A. Polglase, General Surgeon, (Francis Xavier Cabrini Hospital) and associated staff, for providing access to tissue specimens and pathologist's reports, Mr B. Veitch for assistance with immunohistochemical techniques and photography, and the Monash University Anatomy Photography Department.

S.J.P. was supported by an N.H. & M.R.C. Medical Postgraduate Scholarship.

### References

AGAWA, S. & JASS, J.R. (1990). Sialic acid histochemistry and the adenoma-carcinoma sequence in the colorectum. *J. Clin. Pathol.*, **43**, 527.

BARA, J., LOISILLIER, F. & BURTIN, P. (1980). Antigens of gastric and intestinal mucous cells in human colonic tumours. *Br. J. Cancer*, **41**, 209.

BARA, J., LANGUILLE, O., GENDRON, M.C., DAHER, N., MARTIN, E. & BURTIN, P. (1983). Immunohistological study of precancerous mucus modification in human distal colonic polyps. *Cancer Res.*, **43**, 3885.

BARA, J., ANDRE, J., GAUTIER, R. & BURTIN, P. (1984). Abnormal pattern of mucus-associated M1 antigens in histologically normal mucosa adjacent to colonic adenocarcinomas. *Cancer Res.*, **44**, 4040.

BOLAND, C.R. (1987). Mucin histochemistry in colonic polyps and cancer. *Semin. Surg. Oncol.*, **3**, 183.

CANNON-ALBRIGHT, L.A., SKOLNICK, M.H., BISHOP, T., LEE, R.G. & BURT, R.W. (1988). Common inheritance of susceptibility to colonic adenomatous polyps and associated colorectal cancers. *New Eng. J. Med.*, **319**, 533.

COOPER, H.S. & REUTER, V.E. (1983). Peanut lectin-binding sites in polyps of the colon and rectum. *Lab. Invest.*, **49**, 655.

DECAENS, C., GAUTIER, R., BARA, J., DAHER, N., LE PENDU, J. & BURTIN, P. (1983). A new mucin-associated oncofetal antigen, a marker of early carcinogenesis in rat colon. *Cancer Res.*, **48**, 1571.

DESCHNER, E.E. (1990). Kinetics of normal, preneoplastic and neoplastic colonic epithelium. In *Cancer Cells*, (ed.) Moyer, M.P. & Boste, G.H., Acad Press: San Diego.

ENTERLINE, H.T. (1976). Polyps and cancer of the large bowel. *Curr. Top. Pathol.*, **63**, 95.

FEARON, E.R. & VOGELSTEIN, B. (1990). A genetic model for colorectal tumorigenesis. *Cell*, **61**, 759.

FEIZI, T., GOOI, H.C., CHILDS, R.A. & 5 others (1984). Mucin-type glycoproteins, *Biochem. Soc. Transactions*, London, 607th meeting, p. 591.

FILIPE, M.I. (1975). Mucous secretion in rat colonic mucosa during carcinogenesis induced by dimethylhydrazine. A morphological and histochemical study. *Br. J. Cancer*, **32**, 60.

FILIPE, M.I. (1979). Mucins in the human gastrointestinal epithelium: a review. *Invest. Cell Pathol.*, **2**, 195.

FILIPE, M.I., MUGHAL, S. & BUSSEY, H.J. (1980). Patterns of mucus secretion in the colonic epithelium in familial polyposis. *Invest. Cell Pathol.*, **3**, 329.

FILIPE, M.I., SANDEY, A. & MA, J. (1988). Intestinal mucin antigens in ulcerative colitis and their relationship with malignancy. *Human Pathol.* **19**, 671.

FOUTCH, D.O., DIDSARIO, J.A., PARDY, K., MAI, H.D. & MANNE, R.K. (1991). The sentinel hyperplastic polyp: a marker for synchronous neoplasia in the proximal colon. *Am. J. Gastroenterol.*, **86**, 8610.

GOH, H.S. & JASS, J.R. (1987). Correlations of size and mass of colorectal adenomas. *Ann. Acad. Med.*, **16**, 421.

GREAVES, P., FILIPE, M.I., ABBAS, S. & ORMEROD, M.G. (1984). Sialomucins and carcinoembryonic antigen in the evolution of colorectal cancer. *Histopathology*, **8**, 825.

GRODEN, J., THLIVERIS, Q., SAMOWITZ, W. & 20 others (1991). Identification and characterization of the familial adenomatous polyposis coli gene. *Cell*, **66**, 589.

HERTZOG, P.J., MA, J., ROBINSON, H.C., MACKAY, I.R. & LINNANE, A.W. (1991*a*). Oncodevelopmental expression of the human intestinal mucin glycoprotein antigens in gastrointestinal epithelium defined by monoclonal antibodies. *Int. J. Cancer*, **48**, 355.

HERTZOG, P.J., PILBROW, S.J., PEDERSEN, J., POLGLASE, A.L., LAWSON, M. & LINNANE, A.W. (1991*b*). Aberrant expression of intestinal mucin antigens associated with colorectal cancer defined by a panel of monoclonal antibodies and comparison with CEA. *Br. J. Cancer*, **64**, 799.

HILL, M.J., MORSON, B.C., BUSSEY, H.J.R. (1978). Aetiology of adeno-carcinoma sequence in large bowel. *Lancet*, **i**, 245–247.

HOSHINO, Y., HORIKAWA, U., OSHIMURA, M. & YUASA, Y. (1991). Normal human chromosome 5, on which a familial adenomatous polyposis gene is located, has tumor suppressive activity. *Biochem. Biophys. Res. Comm.*, **174**, 298.

ISAACSON, P. & ATTWOOD, P.R.A. (1979). Failure to demonstrate specificity of the morphological and histochemical changes in mucosa adjacent to colonic carcinoma (transitional mucosa). *J. Clin. Pathol.*, **32**, 214.

ITZKOWITZ, S.H., YUAN, M., FERRELL, L.D., PALEKAR, A. & KIM, Y.S. (1986). Cancer-associated alterations of blood group antigen expression in human colorectal polyps. *Cancer Res.*, **46**, 5976.

JACKMAN, R.J. & MAYO, C.W. (1951). The adenoma-carcinoma sequence in cancer of the colon. *Surg. Gynae. Obst.*, **83**, 327.

JASS, J.R. (1983). Relation between metaplastic polyp and carcinoma of the colorectum. *Lancet*, Jan 1/8, 28.

JASS, J.R. & SOBIN, L.H. (1989). Histological typing of intestinal tumors. *WHO International Histological Classification of Tumors*, 2nd Ed, Springer-Verlag, Berlin, 29.

KIM, Y.S., YUAN, M., ITZKOWITZ, S.H. & 5 others (1986). Expression of Le-y and extended Le-y blood group-related antigens in human malignant, premalignant, and nonmalignant colonic tissues. *Cancer Res.*, **46**, 5985.

LISTINSKY, C.M. & RIDDELL, R.H. (1981). Patterns of mucin secretion in neoplastic and non-neoplastic diseases of the colon. *Human. Pathol.*, **12**, 923.

MUGHAL, S. & FILIPE, M.I. (1978). Ultrastructural study of the normal mucosa-adenoma-cancer sequence in the development of familial polyposis coli. *J. Natl Cancer Inst.*, **60**, 753.

MUTO, T., BUSSEY, H.J.R. & MORSON, B.C. (1975). The evolution of cancer of the colon and rectum. *Cancer*, **36**, 2251.

NISHISHO, I., NAKAMURA, Y., MIYOSHI, Y. & 19 others (1991). Mutations of chromosome 5q21 genes in FAP and colorectal cancer patients. *Science*, **253**, 665.

PILBROW, S.J., HERTZOG, P.J. & LINNANE, A.W. (1992*a*). Expression of a novel family of epitopes on small intestinal mucins in colorectal cancer, adjacent and remote mucosa. *Tumor Biol*, (in press).

PILBROW, S.J., HERTZOG, P.J., PINCZOWER, G.D. & LINNANE, A.W. (1992*b*). Expression of large intestinal mucin antigen (LIMA) epitopes in the normal and neoplastic gastrointestinal tract. *J. Pathol.*, (in press).

PODOLSKY, D.K. & FOURNIER, D.A. (1988). Alterations in mucosal content of colonic glycoconjugates in inflammatory bowel disease defined by monoclonal antibodies. *Gastroenterology*, **95**, 379.

PURDIE, C.A., O'GRADY, J., PIRIS, J., WYLLIE, A.H. & BIRD, C.C. (1991). p53 expression in colorectal tumors. *Am. J. Pathol.*, **138**, 807.

RUGGERIO, F., COOPER, H.S. & STEPLEWSKI, Z. (1988). Immunohistochemical study of colorectal adenomas with monoclonal antibodies against blood group antigens (Sialosyl-Le-a, Le-a, Le-a, Le-b, Le-x, Le-y, A, B, and H). *Lab. Invest.*, **59**, 96.

SCHMIDBAUER, G. & HEILMANN, K.L. (1980). Morphology and Histochemistry of the Mucosa surrounding small oligotubular adenomas of the large bowel. *Path. Res. Pract.*, **180**, 45.

SLOMSKI, C.A., DURHAM, J.P. & WATNE, A.L. (1986). Glycosyl transferase levels in Familial Polyposis Coli. *J. Surg. Res.*, **40**, 406.

STRYKER, S.J., WOLFF, B.G., CULP, C.E., LIBBE, S.D., ILSTRUP, D.M. & MACCARTY, R.L. (1987). Natural history of untreated colonic polyps. *Gastroenterology*, **93**, 1009.

SUGIHARA, K. & JASS, J.R. (1987). Colorectal goblet cell mucins in familial adenomatous polyposis. *J. Clin. Pathol.*, **40**, 608.

TEOH, H.H., DELAHUNT, B. & ISBISTER, W.H. (1989). Dysplastic and malignant areas in hyperplastic polyps of the large intestine. *Pathology*, **21**, 138.

VOGELSTEIN, B., FEARON, E.R., HAMILTON, S.R. & 8 others (1988). Genetic alterations during colorectal tumor development. *New Eng. J. Med.*, **319**, 525.

WOLF, B.C., D'EMILIA, J.C., SALEM, R.R. & 4 others (1989). Detection of the tumor-associated glycoprotein antigen (TAG-72) in premalignant lesions of the colon. *J. Natl Cancer Inst.*, **81**, 1913.

ZOTTER, St., LOSSNITZER, A., HAGEMAN, Ph.C., DELEMARRE, J.F.M., HILKENS, J. & HILGERS, J. (1987). Immunohistochemical localization of the epithelial marker MAM-6 in invasive malignancies and highly dysplastic adenomas of the large intestine. *Lab. Invest.*, **57**, 193.


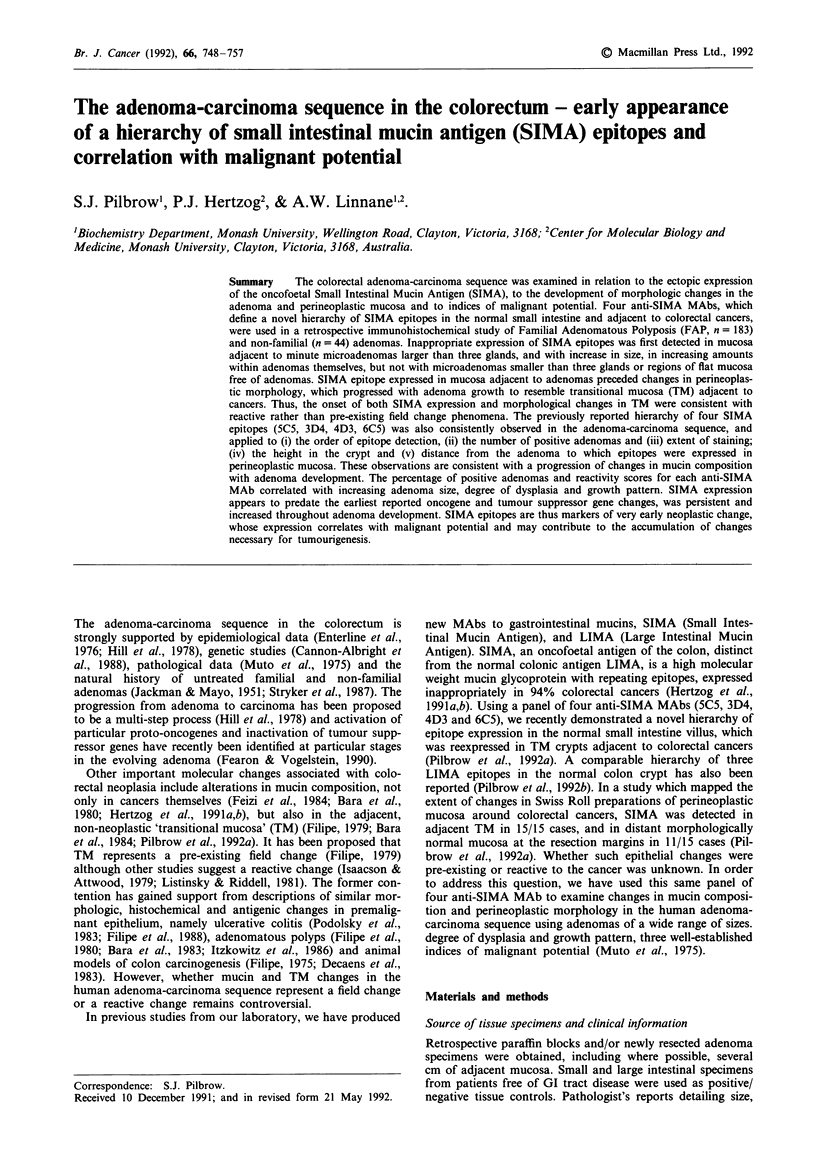

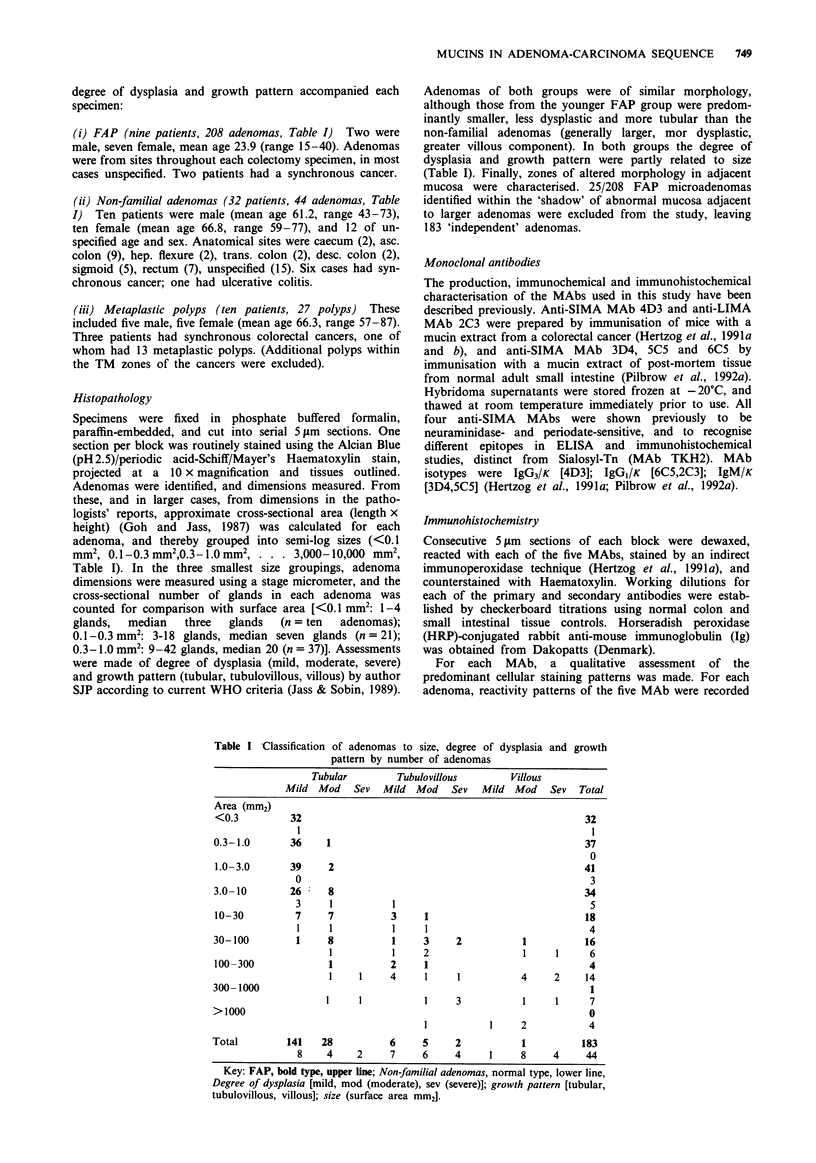

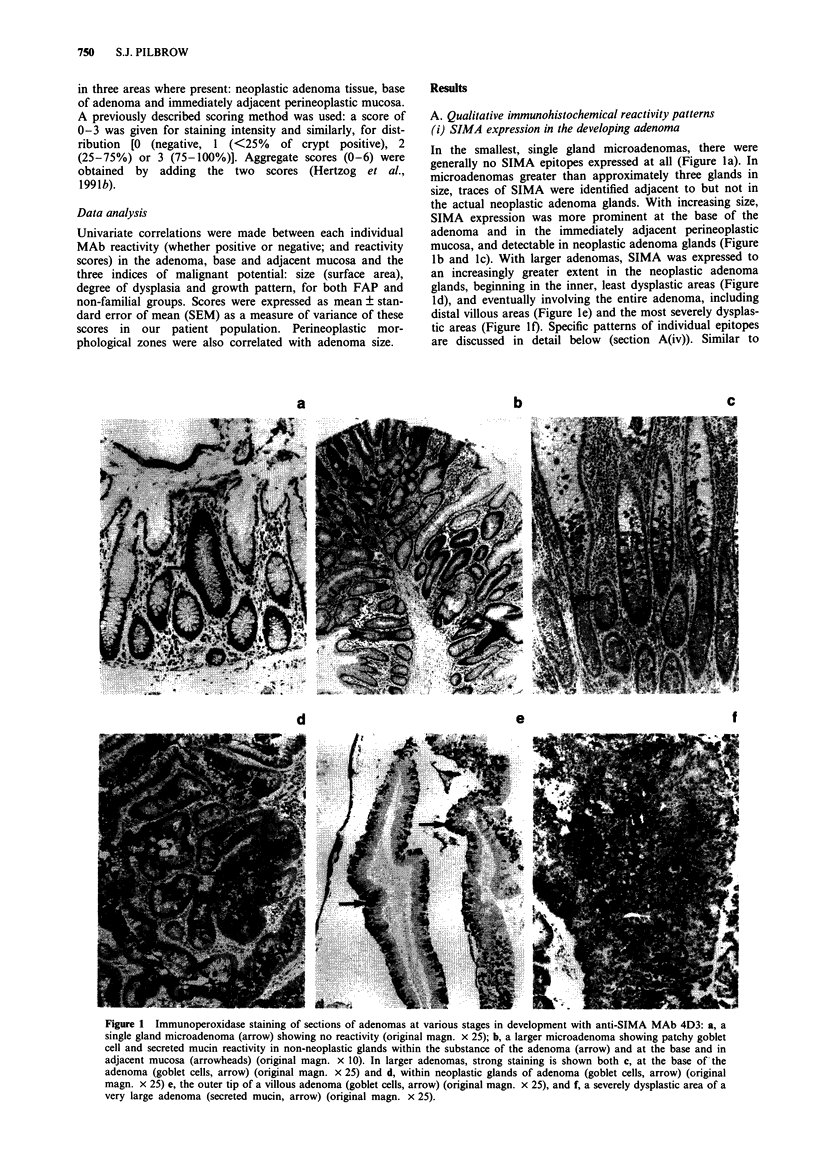

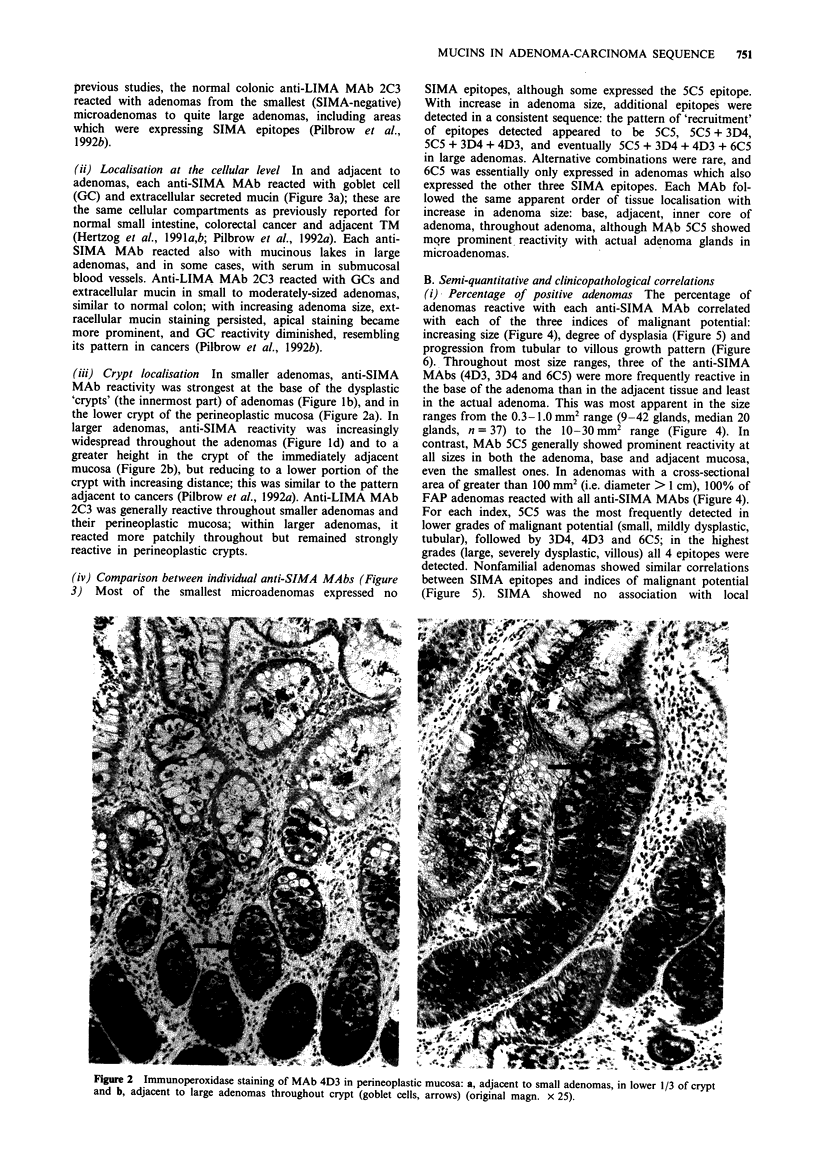

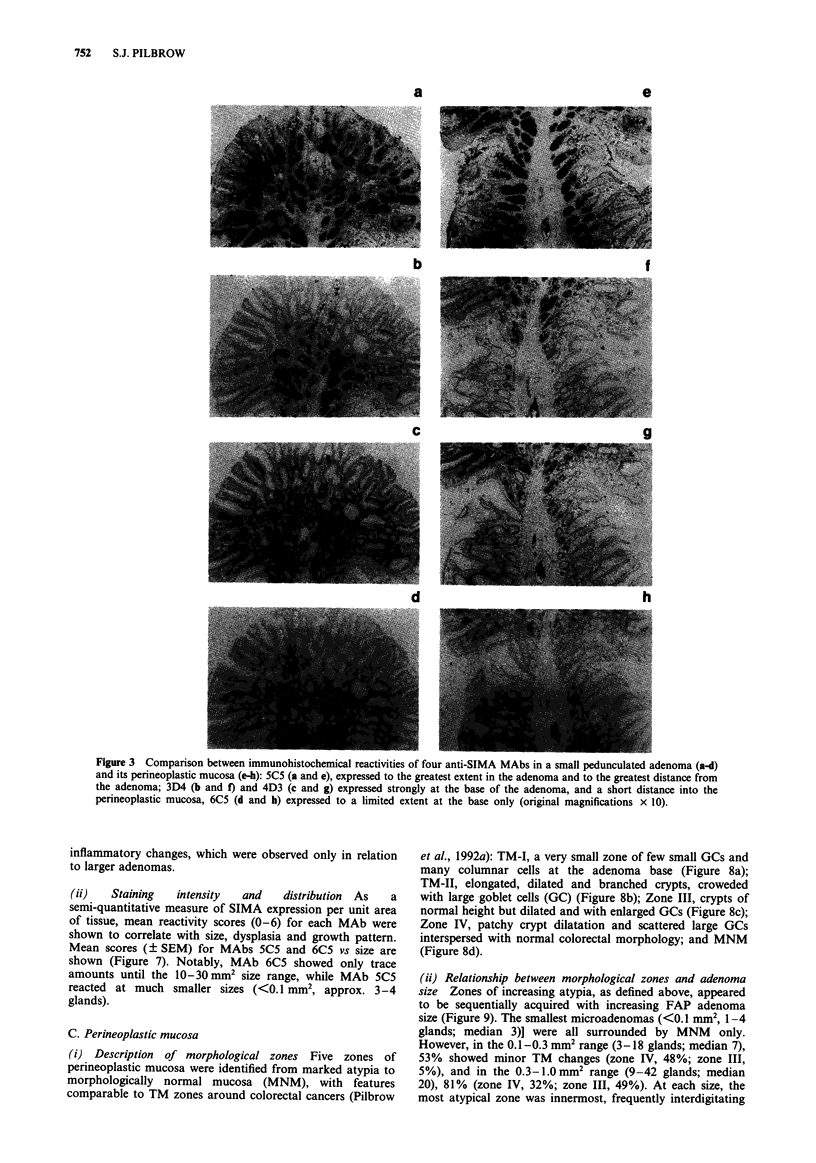

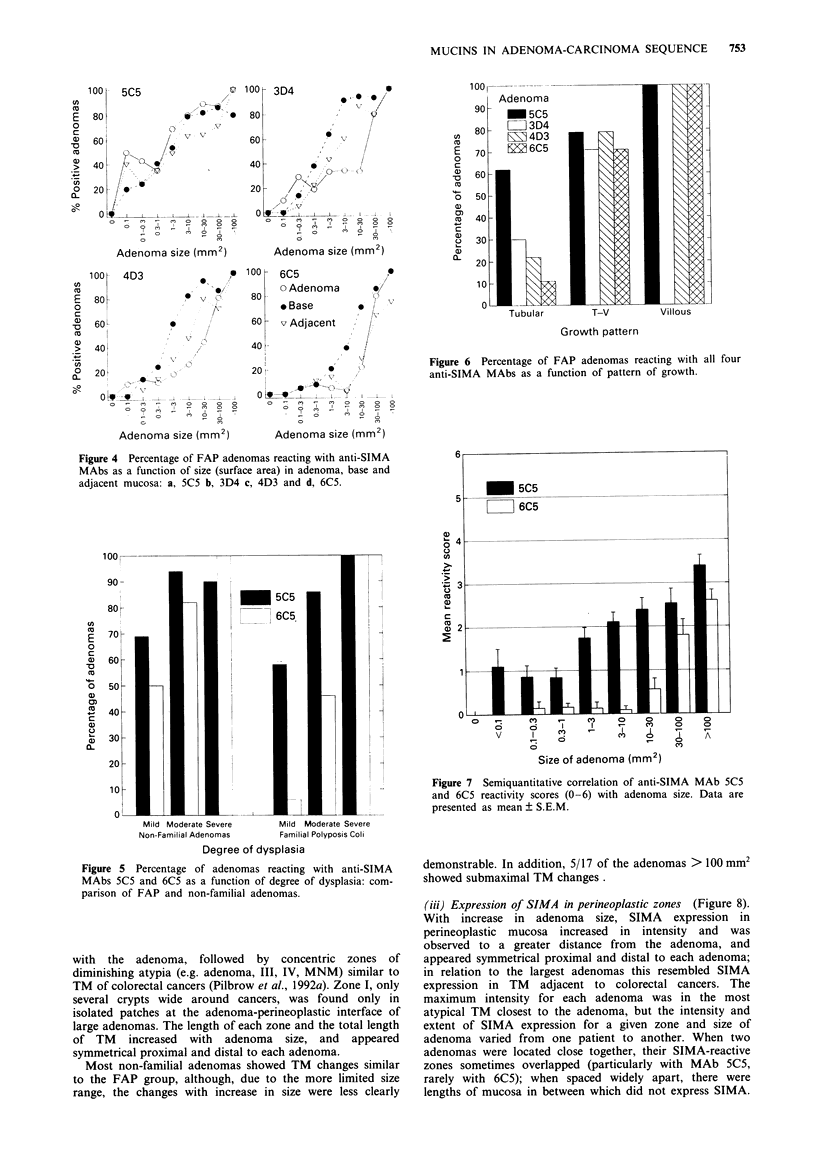

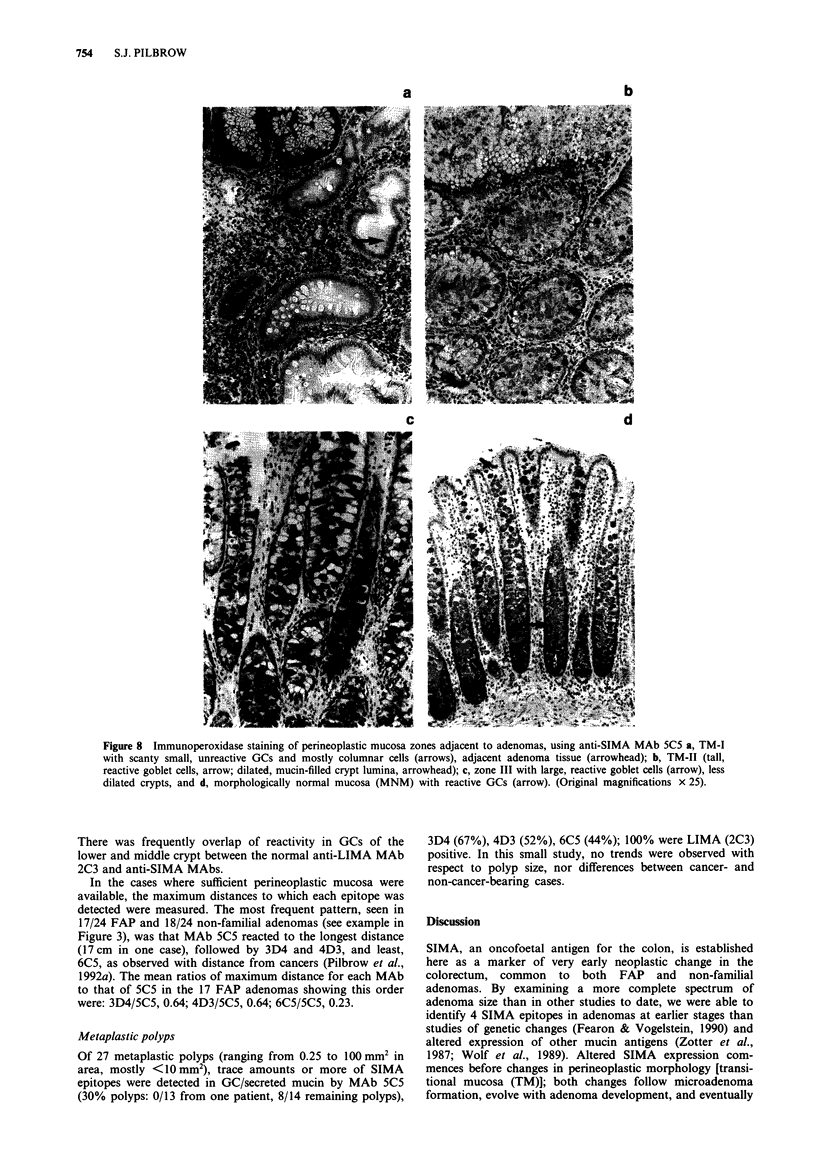

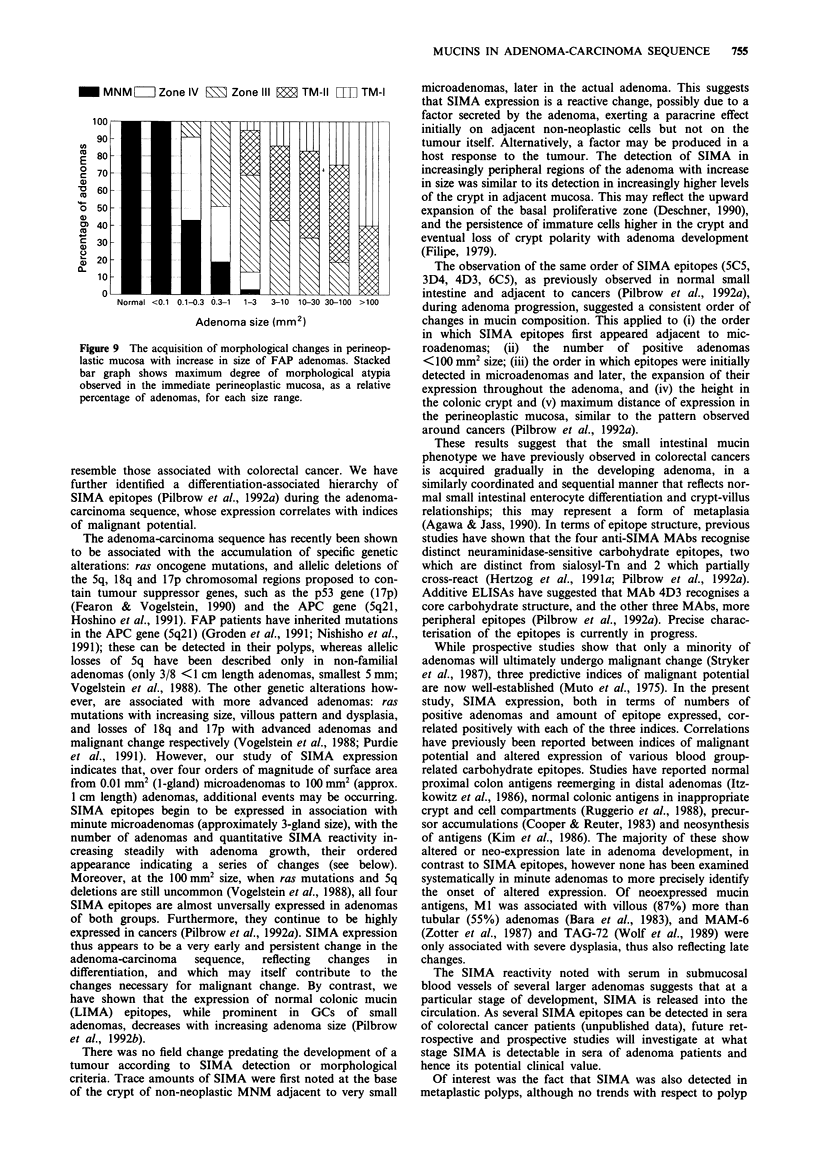

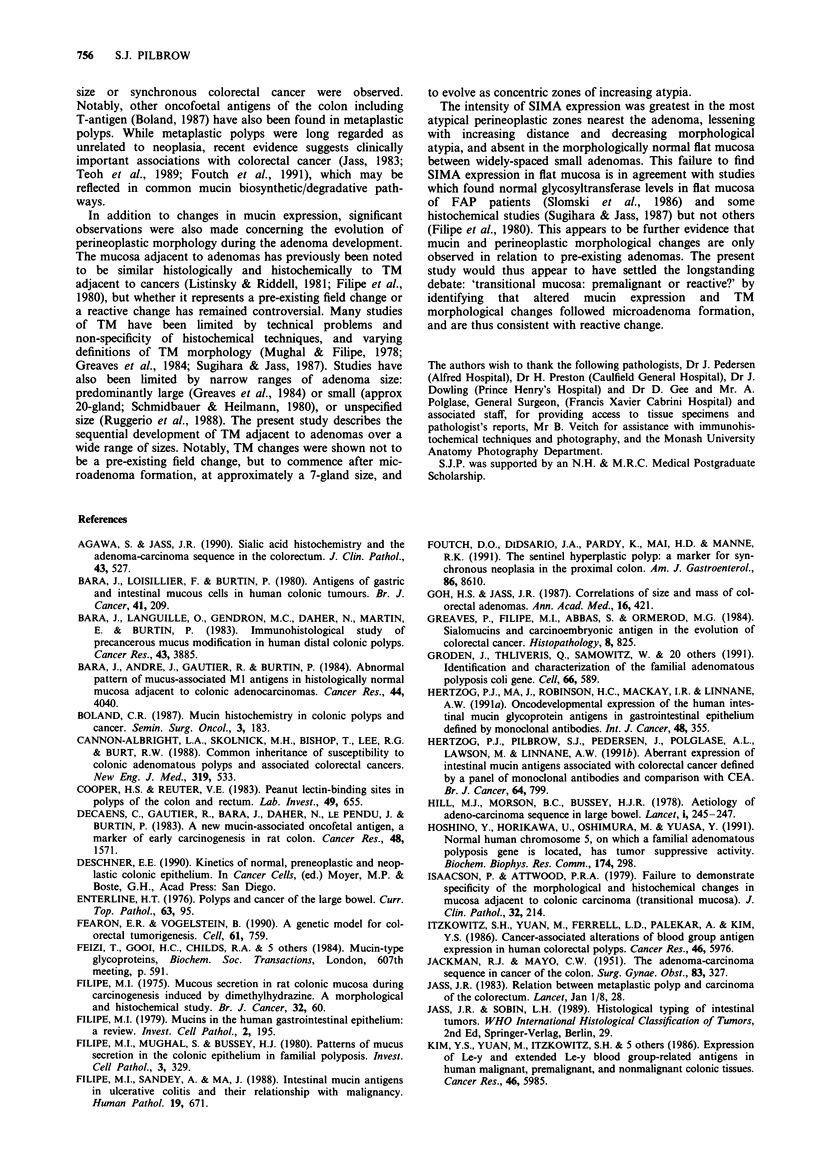

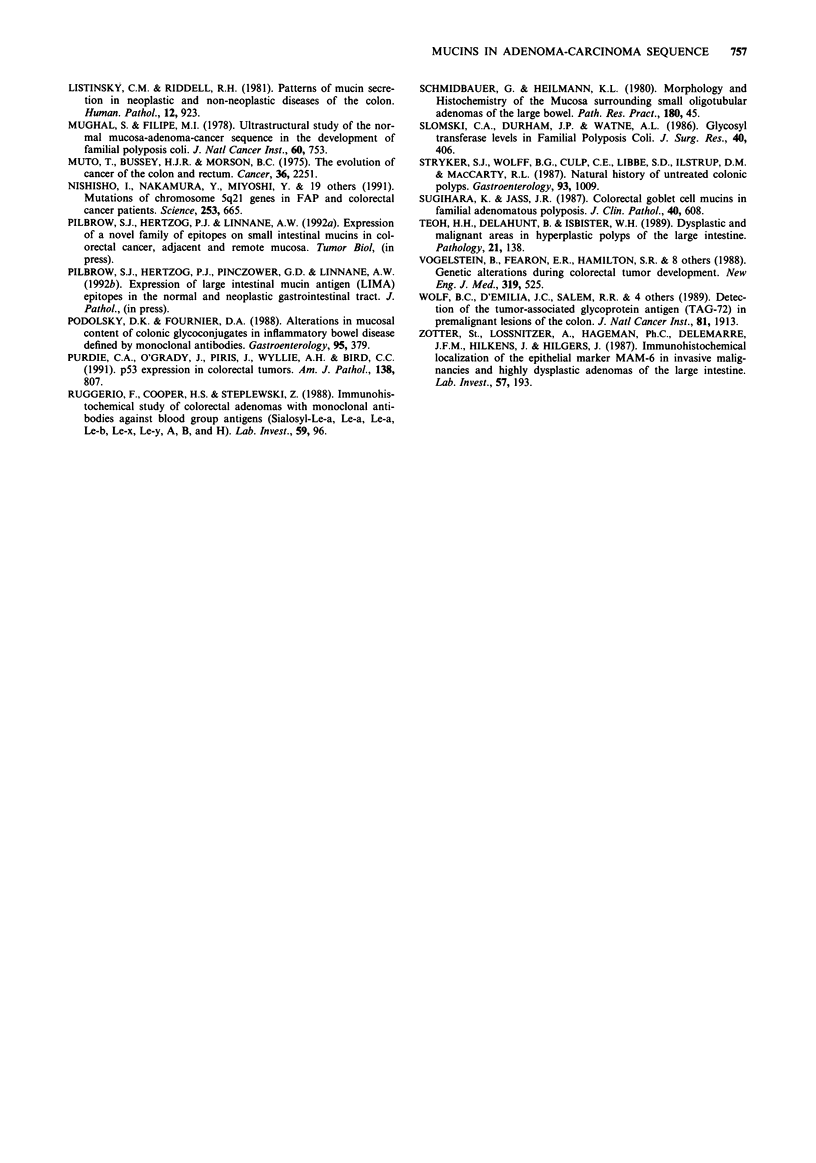


## References

[OCR_01066] Agawa S., Jass J. R. (1990). Sialic acid histochemistry and the adenoma-carcinoma sequence in colorectum.. J Clin Pathol.

[OCR_01082] Bara J., André J., Gautier R., Burtin P. (1984). Abnormal pattern of mucus-associated M1 antigens in histologically normal mucosa adjacent to colonic adenocarcinomas.. Cancer Res.

[OCR_01076] Bara J., Languille O., Gendron M. C., Daher N., Martin E., Burtin P. (1983). Immunohistological study of precancerous mucus modification in human distal colonic polyps.. Cancer Res.

[OCR_01071] Bara J., Loisillier F., Burtin P. (1980). Antigens of gastric and intestinal mucous cells in human colonic tumours.. Br J Cancer.

[OCR_01088] Boland C. R. (1987). Mucin histochemistry in colonic polyps and cancer.. Semin Surg Oncol.

[OCR_01092] Cannon-Albright L. A., Skolnick M. H., Bishop D. T., Lee R. G., Burt R. W. (1988). Common inheritance of susceptibility to colonic adenomatous polyps and associated colorectal cancers.. N Engl J Med.

[OCR_01098] Cooper H. S., Reuter V. E. (1983). Peanut lectin-binding sites in polyps of the colon and rectum. Adenomas, hyperplastic polyps, and adenomas with in situ carcinoma.. Lab Invest.

[OCR_01102] Decaens C., Gautier R., Bara J., Daher N., Le Pendu J., Burtin P. (1988). A new mucin-associated oncofetal antigen, a marker of early carcinogenesis in rat colon.. Cancer Res.

[OCR_01113] Enterline H. T. (1976). Polyps and cancer of the large bowel.. Curr Top Pathol.

[OCR_01117] Fearon E. R., Vogelstein B. (1990). A genetic model for colorectal tumorigenesis.. Cell.

[OCR_01121] Feizi T., Gooi H. C., Childs R. A., Picard J. K., Uemura K., Loomes L. M., Thorpe S. J., Hounsell E. F. (1984). Tumour-associated and differentiation antigens on the carbohydrate moieties of mucin-type glycoproteins.. Biochem Soc Trans.

[OCR_01131] Filipe M. I. (1979). Mucins in the human gastrointestinal epithelium: a review.. Invest Cell Pathol.

[OCR_01126] Filipe M. I. (1975). Mucous secretion in rat colonic mucosa during carcinogenesis induced by dimethylhydrazine. A morphological and histochemical study.. Br J Cancer.

[OCR_01135] Filipe M. I., Mughal S., Bussey H. J. (1980). Patterns of mucus secretion in the colonic epithelium in familial polyposis.. Invest Cell Pathol.

[OCR_01140] Filipe M. I., Sandey A., Ma J. (1988). Intestinal mucin antigens in ulcerative colitis and their relationship with malignancy.. Hum Pathol.

[OCR_01151] Goh H. S., Jass J. R. (1987). Correlations of size and mass of colorectal adenomas.. Ann Acad Med Singapore.

[OCR_01155] Greaves P., Filipe M. I., Abbas S., Ormerod M. G. (1984). Sialomucins and carcinoembryonic antigen in the evolution of colorectal cancer.. Histopathology.

[OCR_01160] Groden J., Thliveris A., Samowitz W., Carlson M., Gelbert L., Albertsen H., Joslyn G., Stevens J., Spirio L., Robertson M. (1991). Identification and characterization of the familial adenomatous polyposis coli gene.. Cell.

[OCR_01171] Hertzog P. J., Pilbrow S. J., Pedersen J., Polglase A. L., Lawson M., Linnane A. W. (1991). Aberrant expression of intestinal mucin antigens associated with colorectal carcinoma defined by a panel of monoclonal antibodies.. Br J Cancer.

[OCR_01165] Hertzog P. J., Robinson H. C., Ma J., Mackay I. R., Linnane A. W. (1991). Oncofetal expression of the human intestinal mucin glycoprotein antigens in gastrointestinal epithelium defined by monoclonal antibodies.. Int J Cancer.

[OCR_01178] Hill M. J., Morson B. C., Bussey H. J. (1978). Aetiology of adenoma--carcinoma sequence in large bowel.. Lancet.

[OCR_01182] Hoshino Y., Horikawa I., Oshimura M., Yuasa Y. (1991). Normal human chromosome 5, on which a familial adenomatous polyposis gene is located, has tumor suppressive activity.. Biochem Biophys Res Commun.

[OCR_01188] Isaacson P., Attwood P. R. (1979). Failure to demonstrate specificity of the morphological and histochemical changes in mucosa adjacent to colonic carcinoma (transitional mucosa).. J Clin Pathol.

[OCR_01194] Itzkowitz S. H., Yuan M., Ferrell L. D., Palekar A., Kim Y. S. (1986). Cancer-associated alterations of blood group antigen expression in human colorectal polyps.. Cancer Res.

[OCR_01199] JACKMAN R. J., MAYO C. W. (1951). The adenoma-carcinoma sequence in cancer of the colon.. Surg Gynecol Obstet.

[OCR_01212] Kim Y. S., Yuan M., Itzkowitz S. H., Sun Q. B., Kaizu T., Palekar A., Trump B. F., Hakomori S. (1986). Expression of LeY and extended LeY blood group-related antigens in human malignant, premalignant, and nonmalignant colonic tissues.. Cancer Res.

[OCR_01220] Listinsky C. M., Riddell R. H. (1981). Patterns of mucin secretion in neoplastic and non-neoplastic diseases of the colon.. Hum Pathol.

[OCR_01225] Mughal S., Filipe M. I. (1978). Ultrastructural study of the normal mucosa-adenoma-cancer sequence in the development of familial polyposis coli.. J Natl Cancer Inst.

[OCR_01230] Muto T., Bussey H. J., Morson B. C. (1975). The evolution of cancer of the colon and rectum.. Cancer.

[OCR_01236] Nishisho I., Nakamura Y., Miyoshi Y., Miki Y., Ando H., Horii A., Koyama K., Utsunomiya J., Baba S., Hedge P. (1991). Mutations of chromosome 5q21 genes in FAP and colorectal cancer patients.. Science.

[OCR_01251] Podolsky D. K., Fournier D. A. (1988). Alterations in mucosal content of colonic glycoconjugates in inflammatory bowel disease defined by monoclonal antibodies.. Gastroenterology.

[OCR_01256] Purdie C. A., O'Grady J., Piris J., Wyllie A. H., Bird C. C. (1991). p53 expression in colorectal tumors.. Am J Pathol.

[OCR_01261] Ruggiero F., Cooper H. S., Steplewski Z. (1988). Immunohistochemical study of colorectal adenomas with monoclonal antibodies against blood group antigens (sialosyl-Le(a), Le(a), Le(b), Le(x), Le(y), A, B, and H).. Lab Invest.

[OCR_01267] Schmidbauer G., Heilmann K. L. (1985). Morphology and histochemistry of the mucosa surrounding small oligotubular adenomas of the large bowel.. Pathol Res Pract.

[OCR_01272] Slomski C. A., Durham J. P., Watne A. L. (1986). Glycosyltransferase levels in familial polyposis coli.. J Surg Res.

[OCR_01277] Stryker S. J., Wolff B. G., Culp C. E., Libbe S. D., Ilstrup D. M., MacCarty R. L. (1987). Natural history of untreated colonic polyps.. Gastroenterology.

[OCR_01282] Sugihara K., Jass J. R. (1987). Colorectal goblet cell mucins in familial adenomatous polyposis.. J Clin Pathol.

[OCR_01286] Teoh H. H., Delahunt B., Isbister W. H. (1989). Dysplastic and malignant areas in hyperplastic polyps of the large intestine.. Pathology.

[OCR_01293] Vogelstein B., Fearon E. R., Hamilton S. R., Kern S. E., Preisinger A. C., Leppert M., Nakamura Y., White R., Smits A. M., Bos J. L. (1988). Genetic alterations during colorectal-tumor development.. N Engl J Med.

[OCR_01296] Wolf B. C., D'Emilia J. C., Salem R. R., DeCoste D., Sears H. F., Gottlieb L. S., Steele G. D. (1989). Detection of the tumor-associated glycoprotein antigen (TAG-72) in premalignant lesions of the colon.. J Natl Cancer Inst.

[OCR_01301] Zotter S., Lossnitzer A., Hageman P. C., Delemarre J. F., Hilkens J., Hilgers J. (1987). Immunohistochemical localization of the epithelial marker MAM-6 in invasive malignancies and highly dysplastic adenomas of the large intestine.. Lab Invest.

